# Supplementary material for: Phytochemical Profiling, Biological Activities, and In Silico Molecular Docking Studies of Causonis trifolia (L.) Mabb. & J.Wen Shoot
Source: Plants (Basel). 2023 Mar 29;12(7):1495. doi: 10.3390/plants12071495 (PMC10097374; doi:10.3390/plants12071495)
Supplement: Supplementary file 1 [file plants-12-01495-s001.zip › plants-2170288-supplementary.pdf]

Table S1. Adequacy of the model tested.

Model	Sequential p-value	Lack of Fit p-value	Adjusted R	Predicted R	
Linear	0.9846	< 0.0001	-0.2168	-0.4107	
2FI	0.9906	< 0.0001	-0.5653	-1.2881	
**Quadratic**	**< 0.0001**	**0.2642**	**0.9849**	**0.9330**	**Suggested**
Cubic	0.2642		0.9892		

Table S2. The result of ANOVA analysis

**Scheme**	**Sum of Squares**	**Degree of freedom**	**Mean Square**	**F-value**	**p-value**	
**Model**	8000.53	9	888.95	116.87	< 0.0001	significant
A-Solvent	87.58	1	87.58	11.51	0.0115	
B-Temperature	2.87	1	2.87	0.3770	0.5586	
C-Extraction time	1.14	1	1.14	0.1499	0.7102	
AB	36.78	1	36.78	4.84	0.0638	
AC	1.96	1	1.96	0.2577	0.6273	
BC	44.36	1	44.36	5.83	0.0464	
A^2	1904.85	1	1904.85	250.42	< 0.0001	
B^2	2123.01	1	2123.01	279.10	< 0.0001	
C^2	2984.19	1	2984.19	392.32	< 0.0001	
**Residual**	53.25	7	7.61			

Lack of Fit	31.59	3	10.53	1.95	0.2642	not significant
Pure Error	21.65	4	5.41			
**Cor Total**	8053.78	16				
**Standard deviation**	2.76					
**Mean**	119.54					
**CV%**	2.31					
**R^2**	0.9934					
**Adjusted R^2**	0.9849					
**Predicted R^2**	0.9330					
**Adequate precision**	25.2369					

Table S3. Binding energy scores calculated by iGEMDOCK 2.1 for 19 phytocompounds against antiinflammatoryantiinflammatory (e.g., Tnf-α and Cox-2), antioxidant (e.g. SOD) and antidiabetic (α -Amylase and Aldo reductase) target proteins. (Tnf-α: Tumor necrosis factor alpha, Cox-2: Cy-clooxygenase 2, SOD: Superoxide dismutases)

Sl. No.	Name of the compound	Target proteins				
		Antiinflammatory		Antioxidant	Antidiabetic	
		**Tnf-α** (Kcal/mol)	**Cox-2** (Kcal/mol)	**Superoxide dismutase** (Kcal/mol)	**α -Amylase** (Kcal/mol)	**Aldo reductase** (Kcal/mol)
18	1,2,4-Metheno-1H-indene, octahydro-1,7a-dimethyl-5-(1-methylethyl)-, [1S-(1α,2α,3aβ.,4 α.,5α,7aβ.,8S*)]	-70.8462	-70.5264	-60.4064	-62.0786	-70.8876
13	1,4-Methano-1H-indene, octahydro-4-methyl-8-methylene-7-(1-methylethyl)-, [1S-(1α,3aβ.,4α,7α,7aβ)]	-68.373	-77.4816	-56.8787	-59.77	-71.0972
3	1,5-Cyclodecadiene, 1,5-dimethyl-8-(1-methylethenyl)-, [S-	-69.0209	-80.3145	-52.3437	-63.2604	-70.9953

	(Z,E)]-					
5	11,11-Dimethyl-spiro [2,9] dodeca-3,7-dien	-60.8135	-74.7328	-54.7702	-61.4609	-64.6676
14	1H-3a,7-Methanoazulene, octahydro-1,9,9-trimethyl-4-methylene-, (1.α.,3aα.,7 α.,8aβ)	-62.6197	-72.0267	-57.8419	-58.2319	-63.3848
4	1H-Cyclopropa[a]naphthalene. 1a,2,3,5,6,7,7a,7b-octahydro-1,1,7,7a-tetramethyl-, [1aR-(1aα,7α,7aα,7bα)]-	-64.9959	-70.2567	-52.0365	-60.9246	-66.3327
11	1-Isopropyl-4,7-dimethyl-1,2,3,5,6,8a-hexahydronaphthale	-64.3723	-74.823	-56.8958	-66.4112	-75.4169

	ne					
	2H-Pyran, tetrahydro-4-methyl-2-(2-methyl-1-propenyl)	-59.2257	-69.2979	-54.6078	-57.9675	-65.3748
12	3H-3a,7-Methanozulene, 2,4,5,6,7,8-hexahydro-1,4,9,9-tetramethyl-, [3aR-(3aα,4β,7α)]	-61.6378	-64.4594	-53.5194	-60.5456	-64.6886
16	3-Methylmannoside	-72.3599	-83.1708	-68.0867	-69.7768	-83.3957
15	3-O-Methyl-d-glucose	-78.693	-90.4112	-74.4558	-78.8684	-97.5636
6	5,7-Dodecadiyne-1,12-diol	-69.7264	-91.2112	-75.9038	-75.5249	-87.9113
1	Caryophyllene	-61.8269	-67.428	-46.6963	-60.2325	-65.3311
8	Ergosta-5,22-dien-3-ol, acetate, (3β,22E)-	-93.0843	-92.0437	-87.7343	-108.844	-115.66

9	Germacrene D	-70.6613	-81.3181	-53.202	-66.6915	-70.7977
10	Humulene	-63.2139	-71.0528	-49.4899	-59.9427	-66.7441
17	Naphthalene, 1,2,4a,5,6,8a-hexahydro-4,7-dimethyl-1-(1-methylethyl)-	-69.9012	-78.3814	-54.6416	-64.1552	-72.1134
2	α-Copaene	-65.7724	-78.902	-57.0262	-63.7201	-73.4357
7	γ-Elemene	-64.4953	-74.5854	-53.0233	-61.3088	-74.8143
